# Prevalence and social support of LGBT medical students in Northeastern Brazil: an epidemiological analysis

**DOI:** 10.1192/j.eurpsy.2026.10456

**Published:** 2026-07-31

**Authors:** M. I. O. de Morais, L. C. Carneiro, A. F. C. Ximenes, L. V. de Araújo, P. F. B. de Araújo, A. B. D. C. Alves, F. O. Barbosa, B. D. A. A. L. Tavares de Melo, E. D. F. Correia, V. L. D. Melo-Neto, D. B. B. Ferreira

**Affiliations:** 1 Universidade de Ciências da Saúde de Alagoas; 2 Universidade Federal de Alagoas, Maceió, Brazil

## Abstract

**Introduction:**

Minority stress theory states that exposure to prejudice and stigma increases vulnerability to mental health problems among LGBT populations. In higher education, and particularly in medical training, these risks intersect with academic stressors that already compromise student well-being. Yet, cultural and contextual factors, such as family cohesion and peer solidarity, can act as protective buffers. In Brazil, epidemiological data on LGBT medical students remain scarce, hindering the development of evidence-based inclusion policies. Assessing prevalence, perceived social support, and awareness of institutional protections is therefore essential to inform interventions in medical schools.

**Objectives:**

To estimate the prevalence of LGBT students and to assess perceived social support and awareness of institutional diversity policies.

**Methods:**

Cross-sectional study conducted in a medical school in Aracaju (Brazil). A total of 142 undergraduates (population=593; calculated minimum=84; eligibility ≥18 years; informed consent obtained) completed a sociodemographic form, the Beck Depression Inventory, the LGBT Campus Climate Scale, and the Perceived Social Support Scale. Analyses included descriptive statistics, Fisher’s exact test/χ² and ANOVA.

**Results:**

Of 142 students, 7 (4.9%) identified as gay/lesbian, 3 (2.1%) as bisexual, and 132 (93.0%) as heterosexual. LGBT students reported higher social support, with 80% strongly agreeing they could rely on friends/family versus 62% of heterosexuals (p=0.041). Mean support scores were higher among LGBT (4.3 versus 3.9, p<0.05). Awareness of institutional policies was low in both groups: only 20% of LGBT and 22.7% of heterosexuals recognized anti-discrimination regulations. No student identified dedicated support centers or regular diversity activities.

**Conclusions:**

Findings show resilience and robust support networks among LGBT students, challenging assumptions of universal vulnerability. Yet, institutional invisibility persists, underscoring the need for diversity policies, faculty training, and structured support services in medical schools. The single-site design, small LGBT subsample, and cross-sectional nature limit generalizability and prevent causal inferences, but results underscore the need for multicenter studies with longitudinal approaches.

**Disclosure of Interest:**

None Declared

